# Study of Medication Reconciliation Process at the Time of Admission in the Patients of a Tertiary Care Teaching Hospital

**DOI:** 10.7759/cureus.71459

**Published:** 2024-10-14

**Authors:** Krushika Domadiya, Bharat Gajjar, Alpa Gor, Bhalendu Vaishnav

**Affiliations:** 1 Pharmacology, Pramukhswami Medical College, Karamsad, Anand, IND; 2 Internal Medicine, Pramukhswami Medical College, Karamsad, Anand, IND

**Keywords:** drug omission, medication discrepancies, medication reconciliation, patient admission, patient’s safety

## Abstract

Introduction: Reconciliation errors are those that are considered harmful or possibly dangerous to patients; in fact, reconciliation errors significantly influence adverse drug events (ADEs) among admitted and discharged patients. Medication reconciliation facilitates the identification and prevention of medication errors and adverse drug events. Worldwide, ongoing medication management is a major source of concern for patient safety. Therefore, this study aims to study medication reconciliation on admission in trauma and emergency care (TEC) at a tertiary care teaching hospital. The objective of our study was to identify discrepancies and medication errors.

Methods: A prospective observational study on medication reconciliation was conducted at the Tertiary Care Teaching Hospital of Shree Krishna Hospital (SKH) in Karamsad, Anand, from February 2023 to February 2024.

Results: The total number of identified discrepancies on admission was 64 in 45 patients. Out of 64 discrepancies, 39 were of adding type, documented as intentional discrepancies and 25 were of omission type, documented as unintentional discrepancies. The majority of those errors were moderate to severe in terms of severity.

Conclusion: The results of this study demonstrate the importance of identifying medication discrepancies in hospital settings and provide evidence for the need to establish medication reconciliation services that would assist healthcare providers in identifying and resolving medication discrepancies.

## Introduction

Worldwide, the process of ongoing medication management is a major source of concern for patient safety [1]. Research indicates that inadequate medication reconciliation contributes to adverse drug events, toxicities, and clinical complications during the admission, transfer, and discharge processes [2].

Medication errors are defined as “errors in the process of prescribing, dispensing, or administering of medications that may cause or lead to inappropriate medication use or patient harm while the medication is in the control of the health care professional, patient or consumer” [3]. One of the main factors endangering patient safety is medication errors. These errors may result in individual health issues and prolongation of hospitalization and may add to their economic burden. At the time of a patient's hospitalization or discharge, many of these errors are the consequence of an inadequate medication review [4].

During care transitions, such as admission, internal transfer, or discharge, medication discrepancies can affect up to 80% of hospitalized patients. Patients’ management may be hampered during these transitions, and unintentional discrepancies may occur, leading to patient harm. To prevent this, the best possible medication history, including home medications, can be taken according to recent changes and compared with prescribers' current medication orders. This process is referred to as ‘medication reconciliation’, a crucial procedure to ensure patient safety and avoid medication errors [5].

As per the World Health Organization (WHO), “Medication reconciliation is the formal process in which health care professionals' partner with patients to ensure accurate and complete medication information transfer at interfaces of care” [6].

The aforementioned issues can largely be averted through medication reconciliation, a systematic process aimed at enhancing the precision of documented medication histories and their utilization during the prescribing process [6]. The WHO urges all of its member nations to support the adoption of the medication reconciliation standard operating procedure by all of their healthcare facilities in an effort to improve patient safety [6]. Medication reconciliation is still not standardized in practice. However, a number of studies have demonstrated the benefits of medication reconciliation [7]. We must first understand the methodology required to identify medication discrepancies before acknowledging that medication reconciliation is a standard procedure. The present study was conducted to investigate medication reconciliation at a tertiary care teaching hospital. Our study aimed to detect discrepancies and medication errors in trauma and emergency care (TEC) during the admission process.

## Materials and methods

This prospective observational study on medication reconciliation was carried out from February 2023 to February 2024 at trauma and emergency care (TEC) of Shree Krishna Hospital (SKH), a tertiary care teaching hospital in Karamsad, Anand, India. The study was approved by the ethics committee of Pramukhswami Medical College, Bhaikaka University, Karamsad, Anand (Ref. No. IEC/BU/143/11/67/2023).

Patients admitted to TEC were randomly selected for the study. A hundred patients were included in this study. Patients who were suffering from terminal illnesses and patients who could not be counseled due to physical or mental constraints were excluded. Prior to the enrollment of patients in the study, their informed written consent was obtained. 

Patient data were collected through interviews and a review of case files at TEC. A comprehensive patient history was obtained, encompassing demographic details, chief complaints, current and past medical history, allergies, family medical history, menstrual history (for female patients), and previous diseases and treatments. Subsequently, the patient's case files were examined to gather information on admission details (admission date and hospital ID), medical diagnosis, and medication records. All this information was meticulously recorded in a specifically designed case record form, serving as a comprehensive data collection tool.

A comparison was conducted between the patient's prescribed medication upon admission and the medications taken prior to admission (at home), obtained through an interview to detect any discrepancies and ascertain their status, particularly during admission.

The identified discrepancies have been categorized into three main groups: intentional, unintentional, and undocumented intentional. Furthermore, they have been subcategorized into three distinct types: omission, commission (addition), and therapeutic duplication. Intentional discrepancies refer to appropriate variances between admission orders, the patient's care plan, their preferences, and the best available medication history. These variances should be clinically justifiable. Undocumented intentional discrepancies occur when a prescriber purposefully makes changes to a medication without adequate documentation. Unintentional discrepancies arise when a medication that the patient was previously taking is mistakenly altered, added, or omitted by the prescriber. Therapeutic duplication is the practice of prescribing multiple medications for the same purpose without clearly distinguishing when one should be used over another.

The severity of medication discrepancies was classified into three categories. Minor discrepancies would not impact the patient's outcome if the medication was omitted. Moderate discrepancies would adversely affect the patient's outcome if they persisted or occurred. Major discrepancies would harm the patient's outcome if they persisted or were experienced. A patient follow-up process has been implemented to assess the effects of discrepancies on the patients.

Data were gathered and analyzed using Microsoft Excel (Microsoft Corporation, Redmond, Washington, USA), encompassing computations for measures such as the mean, standard deviation, percentage, and frequency.

## Results

Based on the predetermined inclusion and exclusion criteria, a total of 100 patients were included in the study. Among these patients, 45 were found to be taking pre-admission medication. Analysis of the data presented in Table 1 reveals that 27 out of the 45 patients (60%) were male, while the remaining 18 (40%) were female.

**Table 1:** Distribution of patients according to gender (n = 45). Data are presented as N (%).

Gender	Frequency (%)
Male	27 (60%)
Female	18 (40%)

In Table 2, the distribution of study participants is presented based on age groups. The largest proportion of participants fell within the 41-60 age group (n = 19, 42.22%), followed by the 61-80 age group (n = 14, 31.11%), the 21-40 age group (n = 08, 17.77%), and those aged ≤ 20, with the smallest representation, along with participants over 80 years old (n = 2, 4.44%). The mean age of the study participants was 53.08 ± 15.55 years.

**Table 2:** Distribution of patients according to age (n = 45). Data are presented as N (%), mean and SD.

Age category (year)	Frequency (%)	Mean ± SD
≤20	2 (4.44%)	53.08 ± 15.55
21-40	8 (17.77%)	
41-60	19 (42.22%)	
61-80	14 (31.11%)	
>80	2 (4.44%)	

**Figure 1:**
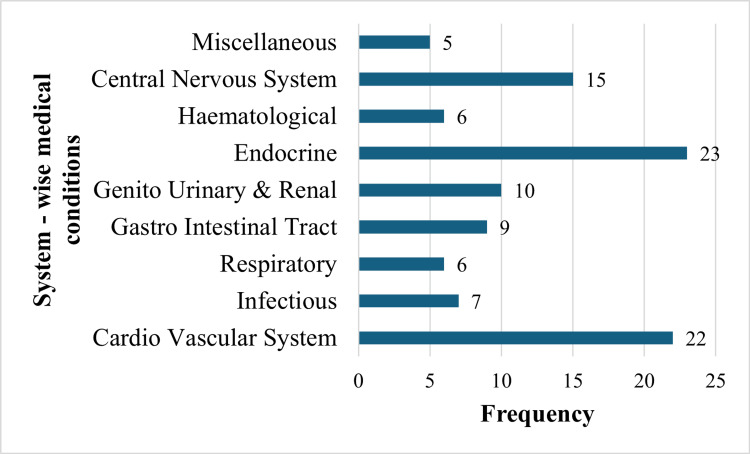
Analysis of study participants as per system-wise medical conditions, including comorbid conditions. Data are presented as frequency.

Figure 1 presents the distribution of study participants based on system-wise medical conditions, including comorbid conditions. The highest number of participants exhibited conditions related to the endocrine system (23) and the cardiovascular system (22). The remaining participants were affected as follows: 15 participants presented conditions related to the central nervous system; 10 participants had genitourinary and renal conditions; nine participants experienced conditions related to the gastrointestinal tract; seven participants were affected by infectious diseases; six participants had hematological conditions; six participants had respiratory conditions; and five participants had miscellaneous conditions, including one participant with hepatobiliary conditions, two participants with ophthalmology conditions, one participant with otorhinolaryngology conditions, and one participant with integumentary conditions.

Table 3 depicts the clinical characteristics of the study participants upon admission. Among the 100 participants, 45 were utilizing pre-admission medication at their residences. Upon admission, 64 discrepancies were identified among 42 participants, resulting in an average of 1.42 discrepancies per participant. Specifically, 20 out of 45 participants exhibited one discrepancy per participant, while 22 participants had two discrepancies per participant.

**Table 3:** Clinical characteristics of study participants on admission (n = 45). Data are presented as N (%). NA: not applicable.

Characteristics	Description	n (%)
Total number of participants	Who takes medicines at home	45 (100%)
Medication	Total number of pre-admission medicine	185 (NA)
Discrepancy status	Yes	42 (93.33%)
	No	3 (6.66%)
Number of discrepancies per participant (1.42)	One discrepancy per participant	20 (31.25%)
	Two discrepancies per participant	22 (68.75%)
Discrepancies	Total number of discrepancies	64 (100%)

**Figure 2:**
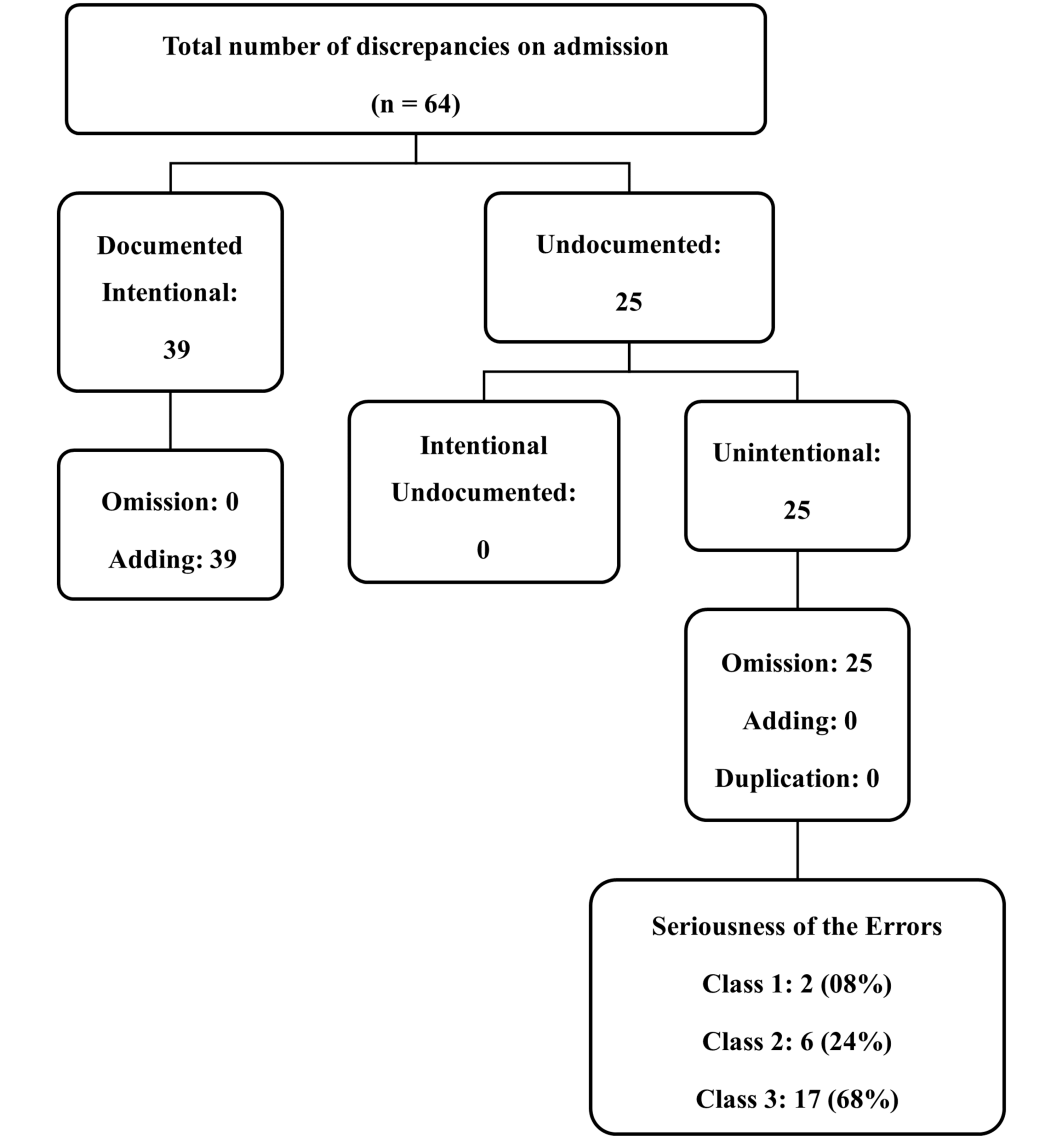
Schematic presentation of medication discrepancies identified among study participants during admission. Data are presented as N (%).

Figure 2 presents the medication discrepancies identified among study participants during admission. A total of 64 discrepancies were identified upon admission. Of these, 39 were documented as intentional, all of which were of the adding type. Additionally, 25 discrepancies were undocumented, and all of these were found to be unintentional omissions. None of the unintentional discrepancies were of the adding or duplication type. Out of 25 unintentional discrepancies, two were classified as minor (class I) errors, which would not impact patient outcomes, and were observed in two patients' prescriptions upon admission. Additionally, three errors were classified as moderate (class II) discrepancies, with the potential to adversely affect patient outcomes if they persisted, and were found in three patients' prescriptions on admission. Furthermore, 17 errors were categorized as major (class III) discrepancies, with the potential to harm patient outcomes if they persisted, and were observed in 17 patients' prescriptions upon admission. Discrepancies, such as intentional, unintentional, and undocumented, were identified in a single prescription at the time of prescribed admission at TEC. These errors were observed across all age groups and were not specific to any particular age group.

**Figure 3:**
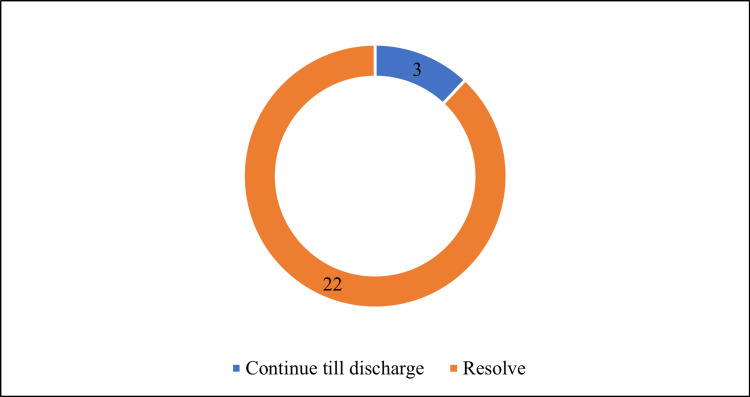
Errors that continued till discharge. Data are presented as N.

In Figure 3, the errors in admission that persisted until discharge are illustrated. Upon transferring patients from the TEC to the ward, 22 admission errors were resolved, with only three errors persisting until discharge.

## Discussion

Medication reconciliation is widely recognized as a vital strategy for improving the quality of medication utilization. Its primary aim is to reduce the incidence of medication discrepancies from the point of hospital admission to discharge [8]. Medication errors may occur during the admission, transfer, or discharge of a patient from the hospital; these transitions appear to pose a particularly high risk for medication errors. According to the World Health Organization (2007), one of the patient safety strategies involves maintaining a precise drug history. Research conducted by Tam et al. revealed that incomplete medication histories during hospital admission could contribute to as much as 37% of hospital prescription errors [9].

The mean age of the participants was 53.08 years with a standard deviation of 15.55, indicating a lower average age compared to other studies, which reported a mean age of 64.91 years with a standard deviation of 18.38 [10].

The mean number of household medications documented in the best possible medical history (BPMH) was 4.1 medications per participant, a figure in close proximity to the study by González‐García et al. (5.55), but lower than the reported average of eight medications per patient by some authors. This variance can be partially attributed to the exclusion of younger patients in the study [10].

We discovered that the cardiovascular and endocrine systems accounted for the majority of admissions. Al-Rashoud et al. reported the most frequent diagnoses at the time of admission were pneumonia (aspiration, community-acquired, or hospital-acquired) (9%), decompensated heart failure (10%), and stroke (11%) [11]. Looking at both setups, it can be said that non-communicable diseases are now taking their high toll. 

The purpose of this study was to identify the different types and frequencies of medication discrepancies observed during hospital admissions at a tertiary care teaching hospital. These discrepancies were detected through medication reconciliation utilizing the best possible medical history (BPMH) approach.

In the conducted study, it was observed that ninety-three percent of participants exhibited discrepancies in their medication. A total of sixty-four medication discrepancies were identified among forty-two participants, attributable to individualized medication adjustments based on their respective medical conditions. Salameh et al.'s research revealed 412 discrepancies among 200 patients [12]. There is considerable variation in the prevalence of medication discrepancies in studies due to the use of different definitions in their assessment and detection. This disparity may be attributed to the varying classifications of medication discrepancies. While some studies categorize medication discrepancies as medication errors, others define them as unintended differences between two lists of medications (i.e., medication lists before and after hospital admission) [12].

This research reveals that, on average, 1.42 discrepancies were identified per participant during the admission process. Climente-Marti et al. reported an average of 4.7 discrepancies per participant, encompassing both admission and discharge discrepancies [13].

In our research, we found that 68.75% of the discrepancies consisted of two per participant, while 31.25% involved one per participant. Contrary to our findings, the study conducted by Alanazi et al. indicates that 58% of the discrepancies were one per participant, 25% involved two per participant, and 17% showed three per participant [14].

In our research, a significant number of discrepancies in medical records, amounting to 60.93%, were found to be intentional as documented by the physician. These discrepancies often arose from new medication orders recommended based on the patient's diagnosis or clinical status. Another study reported a similarly high prevalence of intentional discrepancies, particularly upon admission, reaching 96%. This suggests that medication reconciliation should not simply entail listing all home medications and allowing their continuation without assessing their appropriateness, given the substantial rate of intentional discrepancies [13]. 

Our research revealed that 55.55% of medication discrepancies were of the omission type, while 60.93% were categorized as adding type discrepancies. This contrasts with findings by Mekonnen et al., Alanazi et al., and Mazhar et al., who identified omission as the most frequent cause of medication discrepancy [8,14,15]. In another study, it was found that the most common type of unintentional discrepancy is addition [12].

The presence of undocumented intentional discrepancies poses significant risks, as a prescriber's deliberate modifications without proper documentation can result in confusion and medication errors. This study did not identify any undocumented intentional discrepancies, which contrasts with earlier findings (65%; Salameh et al. [12]). Potential reasons for unrecorded deliberate inconsistencies may stem from team dynamics and the working atmosphere.

The study's discovery of an average of 0.55 (n = 45) unintentional discrepancies per participant upon admission is noteworthy. Previous research by Hellström et al. [16], Okerosi [17], and Vira et al. [18] has shown higher rates of unintentional discrepancies, ranging from 1.5 to 2.3 per patient at the time of hospital admission. Another study by Salameh et al. [12] also reported a similar rate of 0.7 unintentional discrepancies per patient at the time of hospital admission. The potential correlation between this and healthcare organization accreditation merits consideration. Factors such as the utilization of an electronic medical records system [19] at the study site, heightened awareness of patient safety and recovery among medical staff, well-structured reconciliation medical charts, and the participation of hospital physicians in reconciliation workshops are all potential contributors worth noting.

The research consistently highlights omission as the most prevalent unintentional discrepancy in medication reconciliation, with several studies identifying it as the primary issue [16,19-26]. This underscores the significance of addressing omissions when conducting medication reconciliation services. This may be attributed to patients not providing a comprehensive history of their current medications or physicians failing to inquire or document the existing medications in the prescription order.

In this study, the most common unintentional discrepancies were of moderate to major severity, aligning with findings from other studies [12]. Identifying unintentional discrepancies is crucial for alerting medical professionals to the nature of these errors. During the transition of participants from TEC to the ward, 22 out of 25 errors were resolved without intervention. This was attributed to the adherence to medication reconciliation procedures at the time of transfer. Subsequently, only three errors persisted until discharge, and these did not impact the patients' outcomes in terms of hospital stay.

The study was conducted at a single hospital's TEC facility, and the limited sample size may have restricted the generalizability of the findings. However, it is important to acknowledge the significance of the results. One of the study's constraints is the absence of a gold standard for ascertaining home medication use, as we relied on the reports of the patients or their caregivers.

## Conclusions

Upon conclusion, the study revealed a notable prevalence of medication discrepancies among hospitalized patients upon admission to the tertiary care teaching hospital. These discrepancies were associated with a high likelihood of causing moderate to severe harm to the majority of affected patients. Hence, the process of medication reconciliation is an essential component in delivering safe and effective patient care for individuals receiving treatment in a hospital setting, a fact that has been affirmed by this study.
